# The *T*ransitions to *L*ong-term *I*n Home *V*entilator *E*ngagement Study (Transitions to LIVE): study protocol for a pragmatic randomized controlled trial

**DOI:** 10.1186/s13063-022-06035-z

**Published:** 2022-02-07

**Authors:** Reshma Amin, Andrea Gershon, Francine Buchanan, Regina Pizzuti, Adam Qazi, Nishali Patel, Ruxandra Pinto, Myla E. Moretti, Munazzah Ambreen, TtLIVE Group, Paula Abelha, Adele Baker, Sacha Bhatia, Cindy Brennan, Julia Bokhaut, Jackie Chiang, Nisha Cithiravel, Jana Collins, Daniel Cornejo Palma, Leah Costa, Katie N Dainty, Refika Ersu, Tom Fisher, Erin Fleischer, Sandy Fodey, Ian Fraser, Roger Goldstein, Janet Hyatt, Ashley Inman, Mary Irven, Joanna Janevsk, Raj Kohli, Sherri Lynne Katz, Wilma Koopman, Sarah Kuyntjes, David Leasa, Audrey Lim, Paty Sala Lopez, Denise Martins, Cathy Mawdsley, Sandra McKay, Doug McKim, Kristin McMillan, Ramsay McNay, Kevan Mehta, Riley Moss, Jodee Naylor, Mika Nonoyama, Michelle Overholt, April Price, Josee Roy, Madan Roy Christen Shoesmith, Aman Sidhu, Joanne Smith, Lisa Spooner, Aaron St-Laurent, Faiza Syed, Anu Tandon, Mark Thompson, Brenda Toonders, Tuyen Tran, Melissa Trinh, Robert Varadi, Shannon Venance, Kevin Workentin, Allison Zweerink, Louise Rose

**Affiliations:** 1 The Hospital for Sick Children, 555 University Ave, Toronto, M5G 1X8 Canada; 2 Institute for Health Policy, Management, and Evaluation, University of Toronto, 155 College St 4th Floor, Toronto, M5T 3M6 Canada; 3 Child Health and Evaluative Science, SickKids Research Institute, 686 Bay Street, Toronto, Ontario Canada; 4 Sunnybrook Health Sciences Centre, 2075 Bayview Ave, Toronto, M4N 3 M5 Canada; 5 IC/ES, 2075 Bayview Ave, Toronto, M4N 3 M5 Canada; 6 Ontario Child Health Support Unit, The Hospital for Sick Children, 555 University Ave, Toronto, M5G 1X8 Canada; 7 Ontario Ventilator Equipment Pool, Kingston Health Sciences Centre, 640 Cataraqui Woods Dr, Kingston, K7P 2Y5 Canada; 8 Department of Health Metric Sciences, University of Washington, Seattle, WA 98105 USA; 9 Sunnybrook Research Institute, Sunnybrook Health Sciences Centre, 2075 Bayview Ave, Toronto, M4N 3 M5 Canada; 10 Florence Nightingale Faculty of Nursing, Midwifery and Palliative Care, King’s College London, James Clerk Maxwell Building, 57 Waterloo Road, London, SE1 8WA UK; 11 Critical Care Directorate and Lane Fox Respiratory Unit, Guy’s and St Thomas’ NHS Foundation Trust, Westminster Bridge Road, London, SE1 7EH UK

**Keywords:** Mechanical ventilation, Virtual care, eHealth, Telehealth, Home care services, Continuity of patient care, Caregivers, Randomized controlled trial, Cost-utility analysis

## Abstract

**Background overview and rationale:**

We co-developed a multi-component virtual care solution (TtLIVE) for the home mechanical ventilation (HMV) population using the aTouchAway™ platform (Aetonix). The TtLIVE intervention includes (1) virtual home visits; (2) customizable care plans; (3) clinical workflows that incorporate reminders, completion of symptom profiles, and tele-monitoring; and (4) digitally secure communication via messaging, audio, and video calls; (5) Resource library including print and audiovisual material.

**Objectives and brief methods:**

Our primary objective is to evaluate the TtLIVE intervention compared to a usual care control group using an eight-center, pragmatic, parallel-group single-blind (outcome assessors) randomized controlled trial. Eligible patients are children and adults newly transitioning to HMV in Ontario, Canada. Our target sample size is 440 participants (220 each arm). Our co-primary outcomes are a number of emergency department (ED) visits in the 12 months after randomization and change in family caregiver (FC) reported Pearlin Mastery Scale score from baseline to 12 months. Secondary outcomes also measured in the 12 months post randomization include healthcare utilization measured using a hybrid Ambulatory Home Care Record (AHCR-hybrid), FC burden using the Zarit Burden Interview, and health-related quality of life using the EQ-5D. In addition, we will conduct a cost-utility analysis over a 1-year time horizon and measure process outcomes including healthcare provider time using the Care Coordination Measurement Tool. We will use qualitative interviews in a subset of study participants to understand acceptability, barriers, and facilitators to the TtLIVE intervention. We will administer the Family Experiences with Care Coordination (FECC) to interview participants. We will use Poisson regression for a number of ED visits at 12 months. We will use linear regression for the Pearlin Mastery scale score at 12 months. We will adjust for the baseline score to estimate the effect of the intervention on the primary outcomes. Analysis of secondary outcomes will employ regression, causal, and linear mixed modeling. Primary analysis will follow intention-to-treat principles. We have Research Ethics Board approval from SickKids, Children’s Hospital Eastern Ontario, McMaster Children’s Hospital, Children’s Hospital-London Health Sciences, Sunnybrook Hospital, London Health Sciences, West Park Healthcare Centre, and Ottawa Hospital.

**Discussion:**

This pragmatic randomized controlled single-blind trial will determine the effectiveness and cost-effectiveness of the TtLIVE virtual care solution compared to usual care while providing important data on patient and family experience, as well as process measures such as healthcare provider time to deliver the intervention.

**Trial registration:**

ClinicalTrials.gov NCT04180722. Registered on November 27, 2019.

## Administrative information

Note: the numbers in curly brackets in this protocol refer to SPIRIT checklist item numbers. The order of the items has been modified to group similar items (see http://www.equator-network.org/reporting-guidelines/spirit-2013-statement-defining-standard-protocol-items-for-clinical-trials/).

**Table:** 

Title	The **T**ransitions to **L**ong-term **I**n Home **V**entilator **E**ngagement Study (Transitions to LIVE): study protocol for a pragmatic randomised controlled trial
Trial registration {2a and 2b}.	ClinicalTrials.gov: NCT04180722
Protocol version {3}	Version 3; March 16, 2021
Funding {4}	Canadian Institutes of Health Research (CIHR), reference number TC2-165734
Author details {5a}	^1^ The Hospital for Sick Children, 555 University Ave, Toronto, Canada M5G 1X8 ^2^ Institute for Health Policy, Management, and Evaluation, University of Toronto, 155 College St 4th Floor, Toronto, Canada, M5T 3 M6 ^3^ Sunnybrook Health Sciences Centre, 2075 Bayview Ave, Toronto, Canada M4N 3 M5 ^4^ ICES, 2075 Bayview Ave, Toronto, Canada M4N 3 M5 ^5^ Ontario Child Health Support Unit, The Hospital for Sick Children, 555 University Ave, Toronto, Canada M5G 1X8 ^6^ Ontario Ventilator Equipment Pool, Kingston Health Sciences Centre 640 Cataraqui Woods Dr, Kingston, Canada K7P 2Y5 ^7^ Department of Health Metric Sciences, University of Washington, Seattle, WA 98105, United States ^8^ Sunnybrook Research Institute, Sunnybrook Health Sciences Centre, 2075 Bayview Ave, Toronto, Canada M4N 3 M5 ^9^ Florence Nightingale Faculty of Nursing, Midwifery and Palliative Care, King’s College London, James Clerk Maxwell Building, 57 Waterloo Road, London, United Kingdom, SE1 8WA ^10^ Critical Care Directorate and Lane Fox Respiratory Unit, Guy's and St Thomas’ NHS Foundation Trust, Westminster Bridge Road, London, United Kingdom, SE1 7EH
Name and contact information for the trial sponsor {5b}	Ramune Pleinys The Hospital for Sick Children, Toronto Ontario 555 University Ave, Toronto, Canada M5G 1X8
Role of sponsor {5c}	The role of the sponsor includes institutional indemnity insurance for the trial but does not include funding or conduct of the trial.
Composition, Roles and Responsibilities {5d}	The core study team (PI: RA) and two research coordinators will run the trial on a daily basis. The broader research team including site PIs, research team members, local HMV members, and patient and family stakeholders meet monthly at investigator meetings and ad hoc as needed for site specific meetings. These meetings are to provide study updates, troubleshoot issues, and support ongoing engagement of the team. The Trial Steering Committee will be meet twice a year during the study period.

## Introduction

### Background and rationale {6a}

Ventilator-assisted individuals (VAIs) living at home represent a small yet rapidly growing population in Canada [1, 2], and indeed internationally [3, 4]. Canadian prevalence of VAIs is conservatively estimated at 12.9 per 100,000 population [5]. VAIs are among the highest cost users of healthcare resources: a 2018 cost analysis of Canadian adults using Home Mechanical Ventilation (HMV) identified a median (range) monthly cost per VAI of $5275 ($2291–$10,181) with 58% of costs derived from public funding and 39% associated with family caregiving time costs [6]. Beyond economic considerations, HMV places a significant physical, social, and psychological burden on patients and their family caregivers. Communication gaps between and among healthcare providers, patients, and their family caregivers are common. This impedes the prompt resolution of ventilation issues resulting in unnecessary emergent healthcare utilization and costs [7]. Furthermore, the loss of access to trusted healthcare providers following hospital discharge is experienced as service fragmentation [8, 9]. This often leads to the intensification of the family caregiver role. Caring for a VAI at home can negatively affect family caregiver health [10–12]. Infrastructure to support patients and family caregivers is paramount to ensure that VAIs can continue to remain at home, and with substantial cost savings for the public healthcare system.

Virtual care technology provides an opportunity to increase patient (and family) empowerment, enable patient- and family-centered care, facilitate knowledge sharing between healthcare providers and across healthcare sectors, and may eliminate the care silos and negative experiences of transitions across settings experienced by VAIs [13]*.* VAIs are an ideal population for virtual care technology for several reasons. First, clinical follow-up is challenging because of significant costs associated with specialized assistance for travel to healthcare appointments given VAI limited mobility, medical fragility, and reliance on medical technology. VAIs are at risk for adverse events during travel due to inability to maintain access to technology (e.g., pulmonary clearance regimens including timely inhalations and frequent suctioning). This is particularly worrisome as many Canadian VAIs have to travel upwards of 100 km to access the nearest specialist ventilation center [14]. Second, VAIs experience multiple care transitions between and within healthcare sectors as their health status and needs change. Formalized handover between providers is frequently lacking, resulting in information gaps and additional time spent by healthcare providers searching for care plan documentation. Third, VAIs experience a lack of timely access to respiratory health professionals for home follow-up, particularly in the early stages of home transition. International evidence suggests that virtual care has the potential to reduce VAI healthcare utilization and costs to the public health system [15–23].

To our knowledge, no previous study has rigorously evaluated the effectiveness of virtual care as a means of improving health service delivery during the transition to HMV for adults and children. Our *T*ransitions *t*o *L*ong-Term *I*n-Home *V*entilator *E*ngagement (TtLIVE) randomized controlled trial therefore seeks to inform the evidence base by evaluating a multi-component virtual care solution (TtLIVE) for the HMV population using the aTouchAway™ platform (Aetonix, Ottawa, Canada). The TtLIVE intervention enables virtual home visits; customizable care plans; basic clinical workflows that incorporate reminders, completion of symptom profiles and tele-monitoring; digitally secure communication via text messaging, audio and unscheduled video calls between patients, families, and healthcare providers as well as access to an education resource library for patients and families.

### Objectives {7}

We used the Quadruple Aim framework [24], developed by the Institute for Health Care Improvement, as our outcome framework. We focused our study outcomes on improvement in four core domains: (1) health, (2) patient and family experience, (3) health system cost, and (4) healthcare provider time and experience.

Our co-primary objective is to evaluate the effect of the TtLIVE intervention compared to standard of care on (1) emergency department (ED) presentation rates in the 12 months following newly transitioning to HMV and (2) family caregiver reported sense of mastery at 12 months [25].

Secondary objectives are to evaluate (1) number of hospital admissions and days in hospital within 6 and 12 months of newly transitioning to HMV; (2) hospital free survival at 6 and 12 months; (3) time to first ED visit and first hospital admission; (4) respiratory and non-respiratory mortality within 6 and 12 months; (5) number and type of outpatient specialist visits within 6 and 12 months; (6) number of family physician visits within 6 and 12 months; (7) homecare service use within 6 and 12 months; (8) incremental cost of the TtLIVE intervention per patient quality-adjusted life year (QALY) gained compared to usual care from both a health system and societal perspective at 12 months; (9) healthcare provider time using the Care Coordination Measurement Tool; (10) change in family caregiver burden Zarit Burden Interview score from baseline to 6 and 12 months; (11) change in VAI health-related quality of life (HrQoL) using the EQ-5D (adults) and ED-5DY (children) from baseline to 6 and 12 months; (12) change in patient reported Sense of Mastery using the Pearlin Self-Mastery Scale at 12 months; and (13) adverse events.

Process measures include a measure of the quality of care coordination using the Family Experiences of Care Coordination (FECC) measure and through qualitative interviews, as well as fidelity metrics of use of the virtual intervention by patients/families and health care providers.

### Trial design {8}

This will be a superiority trial design. This is a pragmatic parallel group (single blind – outcome assessors) randomized controlled trial (NCT04180722) with a nested qualitative evaluation of the 12-month TtLIVE intervention compared to standard of care, with 1:1 allocation of eligible individuals (children and adults) newly transitioning to HMV. The trial was designed in accordance with the established *Standard Protocol Items: Recommendations for Interventional Trials (SPIRIT) guidance* [26]. For participants allocated to the intervention group, the TtLIVE intervention will be delivered through the aTouchAway™ e-platform (via an electronic tablet, Smartphone, or lap/desktop) by a participating specialist HMV program or clinic. For participants allocated to the control group, usual care of their specialist HMV program or clinic will be provided.

## Methods: participants, interventions, and outcomes

### Study setting {9}

This trial will be conducted in 8 HMV programs/clinics in Ontario, Canada. These 8 centers prescribe ≥ 80% of home ventilators to more than 1200 newly transitioning VAIs annually in the province of Ontario, Canada. Adult centers comprise West Park Healthcare Centre, The Ottawa Hospital Rehabilitation Centre, Sunnybrook Health Sciences Centre, and London Health Sciences Centre. Pediatric centers include The Hospital for Sick Children (SickKids), Children’s Hospital of Eastern Ontario (CHEO), McMaster’s Children’s Hospital, and Children’s Hospital, London Health Sciences. The Ontario Ventilator Equipment Pool is funded by the Ministry of Health and Long-term Care in Ontario and provides ventilators on loan to all children and adults in the province of Ontario receiving HMV.

### Eligibility criteria {10}

Inclusion and exclusion criteria for the trial are detailed in Tables 1 and 2.

**Table 1:** Inclusion and exclusion criteria for study participants

Inclusion	Exclusion
• Newly initiated (in-hospital or outpatient), defined as within 2 months, on a ventilator for HMV prescribed by a participating clinic	• Projected life expectancy of ≤ 2 months
• Reads, writes, and understands English. If patient does not meet this criterion, they have a caregiver that does	• Significant cognitive impairment and absence/inability of a family caregiver able to use aTouchAway™ or complete questionnaires on the subject’s behalf
• Provides informed consent	• Uncontrolled psychiatric illness
• Live in a non-institutionalized setting	• Enrolled in a research study to evaluate another eHealth platform or care coordination model of care
	• Plan to move outside of province in the next 12 months

**Table 2:** Inclusion and exclusion criteria for family caregiver participants

Inclusion	Exclusion
• Primary caregiver of an individual newly initiated on HMV that consents to participation	N/A
• Reads, writes, and understands English	
• Provides informed consent	

### Additional consent provisions for collection and use of participant data and biological specimens {26b}

This trial does not involve collecting biological specimens for storage.

## Interventions

### Explanation for the choice of comparators {6b}

In this trial, control arm participants will receive the current standard of clinical care. Care will be delivered in accordance with the Canadian Thoracic Society (CTS) HMV clinical practice guidelines and include scheduled face-to-face or virtual (due to the COVID-19 pandemic) clinic visits with the HMV team. Additional telephone calls and emails for equipment troubleshooting and management of intercurrent illnesses will depend on medical stability. Other recommended practices include sleep studies and spirometry (where feasible) with frequency based on disease diagnosis and severity; access to the Ontario Ventilator Equipment Pool (VEP) 24-h hotline for ventilator equipment-related issues; an action plan for acute respiratory infection and/or deterioration/disease progression; and a troubleshooting plan for ventilator-related issues. In addition, all patients are offered remote ventilator monitoring.

Proactive bi-monthly monitoring of symptoms is not standard of care at the eight participating study sites where care outside of scheduled clinic visits is reactive and in response to VAI and family caregiver-raised causes for concern. VAIs or family caregivers can contact a member of the HMV team at each of the eight study sites outside of scheduled clinic visits during business hours for assistance with such issues. After business hours clinical issues and emergencies are directed to the ED.

### Intervention description {11a}

Each intervention group participant will receive the TtLIVE intervention delivered through the aTouchAway™ e-platform (Aetonix, Ottawa, Canada). TtLIVE comprises five main components: scheduled virtual clinic visits, virtual care and action plans, bimonthly and monthly monitoring of symptoms, monitoring of ventilator usage and related issues, and as needed virtual consultation and education resources (Table 3).

**Table 3:** TtLIVE components

Intervention feature	Details
**Scheduled virtual clinic visits**	I. Virtual clinic visits with VAI, family, and HMV clinical team over the aTouchAway™ platform will be scheduled at the usual frequency of face-to-face or virtual clinic visits in the standard of care group. II. Structure and content will be based on the structure and content of usual care face-to-face or virtual ventilator clinic visit and include clinical history and symptoms, ventilator data download reports, airway clearance device data downloads, and ventilator alarm review and plan of care.
**Virtual care plan and action plan**	I. Co-developed by and accessible to VAI, family, and nominated circle of care members II. Summary of medical diagnoses, medications, allergies, ventilator and cough assist settings and alarms, and special precautions III. Bespoke action plan for clinical and equipment-related issues developed by each clinical team as part of standard clinical care
**Scheduled monitoring**	I. Bimonthly monitoring with the VentSS questionnaire of symptoms/signs, ventilator usage, and/or ventilator-related equipment issues and/or alarms indicative of respiratory infection and/or deterioration/ disease progression II. Programmed daily and/or weekly reminders to encourage adherence with equipment use and/or maintenance III. Monthly monitoring with the S^3^-NIV questionnaire of symptoms/signs, ventilator usage, and/or ventilator-related equipment issues IV. Remote monitoring of ventilators
**Scheduled as needed Consultation** over the aTouchAway™ platform	I. Triggered by concerning symptoms and/or ventilator parameters (ie yellow or red monitoring alerts) II. Requested by VAI/family and/or healthcare providers III. Secure messaging, voice call, virtual or face-to-face visit
**Education resource library**	I. Access to documents and videos customized to their technology type. For example, ventilator cleaning guides, education refreshers on equipment, and methods for troubleshooting ventilator problems.

#### Intervention delivery

*Scheduled virtual clinic visits* will take place during the first week of trial enrolment and then at 3 months (±2 weeks), 6 months (±2 weeks), 9 months (±2 weeks), and 12 months (±2 weeks) depending on medical stability. Visit frequency aligns with usual care in-person or virtual visits.

A virtual care plan will be completed in partnership with the participant, caregiver, and health care team. This will include a summary of medical diagnoses, medications, allergies, ventilator and cough assist settings, and alarms as well as special precautions. The healthcare providers that are within the circle of care for the participant, will be given permission to access their “virtual chart.” In addition, during the first visit, a *virtual action plan* will be developed by the HMV team in partnership with the participant and caregiver to address clinical and/or ventilator-related concerns. This care plan will include triggers for plan activation and actions in the event of respiratory infection/deterioration and ventilator alarm issues. A pre-determined calling tree is embedded to enable timely and appropriate response from the appropriate responder for medical and ventilator concerns.

*Routine monitoring* of clinical symptoms, ventilator usage, and other equipment-related issues will be conducted through participant bimonthly (VentSS) and monthly (S^3^-NIV) questionnaires administered and answered over the aTouchAway™ platform. We have selected the S^3^-NIV questionnaire, developed for telemonitoring of patients using NIV [25], as our monthly questionnaire. We have included an adapted version for the study participants using invasive ventilation.

*As needed scheduled clinical consultations* can be requested by the VAI, caregiver, and/or healthcare providers in the event of (1) abnormal parameters detected from routine data monitoring (i.e., questionnaire responses yield yellow or red notifications to the healthcare team); and (2) requests made by the VAI or caregiver. We have programmed questionnaire responses within the aTouchaway platform so that one response of concern results in a yellow notification and > 1 results in a red notification.

#### Intervention training

VAIs and their family members will be able to access aTouchAway™ on a range of devices including laptops, desktops, tablets, and Smartphones. Participants without access to a reliable smart device and internet will be offered a study tablet. Prior to distributing the tablet, the research team will create a secure, encrypted account for that VAI. VAIs and the families will receive formalized training (onboarding) on the TtLIVE intervention (via home or virtual visit) by a member of the research team. Instruction will be given on how to (1) operate and navigate the aTouchAway™ platform; (2) complete the virtual care plan; (3) initiate a telephone/ videoconference call; and (4) how to document and upload the ventilator and symptom monitoring data to the ventilator team.

Healthcare providers will use computers and/or smartphones enabled with aTouchAway™ to access the TtLIVE intervention. A research team member will train healthcare providers on the process of downloading the platform onto their device, creating their account, and adding them to the circle of care for a VAI.

### Criteria for discontinuing or modifying allocated interventions {11b}

The TtLIVE intervention would be discontinued if requested by a participant.

### Strategies to improve adherence to the intervention {11c}

A multimodal strategy will be employed to enhance TtLIVE intervention adherence. Study participants that do not complete their symptom and ventilator surveys within an a priori established period of 24 h or 48 h will receive two reminders via the aTouchAway™ app for each questionnaire needing completion. Intervention participants will also have access to the unblinded research coordinator through secure messaging, phone, or video call to troubleshoot any issues, review the app features, or answer any questions.

### Relevant concomitant care permitted or prohibited during the trial {11d}

Co-enrolment in another research study involving an eHealth intervention or care coordination model of care will be prohibited for the duration of the study period.

### Provisions for post-trial care {30}

Towards the end of the 12-month study period, the local HMV team will prepare the patients, caregivers, and healthcare providers for transition back to usual care for study participants allocated to the intervention group.

### Outcomes {12}

See Table 4 for a list of study outcomes.

**Table 4:** Study outcomes and study measures/instruments

Study outcomes	Measure/instrument
Co-primary outcomes	
ED visit rates at 12 months	Health administrative data and AHCR-hybrid
Change in family caregiver reported sense of mastery at 12 months	Pearlin Self-Mastery Scale score
Secondary outcomes	
Healthcare utilization outcomes	
Number of hospital admissions and days in hospital over 6 and 12 months	Health administrative data and AHCR-hybrid
Hospital free survival at 6 and 12 months	Health administrative data and AHCR-hybrid
Time to first ED visit and first hospital admission	Health administrative data and AHCR-hybrid
Overall survival at 6 and 12 months	Health administrative data and AHCR-hybrid
Number and type of outpatient specialist visits at 6 and 12 months	Health administrative data and AHCR-hybrid
Number of family physician visits at 6 and 12 months	Health administrative data and AHCR-hybrid
Homecare service use at 6 and 12 months	Health administrative data and AHCR-hybrid
Participant and caregiver outcomes	
Change in caregiver burden from baseline to 6 and 12 months	Zarit Burden Interview
Change in VAI health-related quality of life (HrQoL) from baseline to 6 and 12 months	EQ-5D-5L (adults) and EQ-5DY (children)
Change in patient reported sense of mastery at 12 months	Pearlin Self-Mastery Scale score
Ventilator use/alarms and signs and symptoms questionnaire	VentSS and the S^3^-NIV questionnaires
Economic outcomes	
Cost utility (ICER) of TtLIVE intervention compared to usual care in improving patient utility	AHCR-hybrid; provincial datasets (IC/ES); provincial costing sources (OCCI); EQ-5D-5L (adults) and EQ-5DY (children)
Healthcare provider outcomes	
Healthcare provider time	Care Coordination Measurement Tool
Process measure outcomes	
Quality of care coordination	Family Experiences of Care Coordination
Adherence to TtLIVE intervention by VAIs/family caregivers over 12 months and platform usage	aTouchAway Metrics
Adherence to TtLIVE intervention by healthcare providers over 12 months and platform usage	aTouchAway Metrics
Adverse events	
Adverse events unique to the use of the aTouchAway™ platform and the internet (technical issues due to software (aTouchAway™ platform) or hardware (the iPad/ phone/ computer being used) failure, privacy, and security breach	Direct reporting from Aetonix, patient, and caregiver

### Data collection

#### Baseline data

We will collect baseline demographic, medical, and psychosocial data on study enrolment. An unblinded Research Coordinator will contact participants via telephone to collect this and notify participants of study allocation following consent and randomization.

##### VAI demographics and characteristics

We will document demographic, medical, and psychosocial characteristics that include smoking status/history and number of pack years (teens and adults); influenza, pneumovax vaccination and COVID-19 status; other medical technology at home; number of hours and type of homecare provider support; and distance (km) from referral ventilation center. We will collect participant’s health card number (Ontario Health Insurance Plan (OHIP) number), date of birth, sex, and randomization allocation for linking to health care databases that contain information about physician, hospital, home care services, and medications that are paid for through universal health insurance administered by the Ontario government.

##### Caregiver demographics and characteristics

The following demographic characteristics will be obtained from the primary caregiver: age, sex, marital status, family income, the highest level of education, and employment status.

#### Healthcare utilization and costs

##### Health administrative data

We will use provincial health administrative data (facilitated through Institute of Clinical Evaluative Science [IC/ES]), collected by the province of Ontario for the administration of its universal health care system, to obtain data on health utilization outcomes over the 12-month trial period. We will verify other public healthcare utilization through participant self-report using the AHCR-hybrid.

Seven provincial health administrative databases will be used: the Ontario Health Insurance Plan Physicians Services Database for information about physician visits; the Canadian Institute of Health Information (CIHI) Discharge Abstract Database for information on hospital admissions; the CIHI National Ambulatory Care Reporting System for information about emergency department visits and ambulatory day surgeries, the Registered Persons Database for demographic information and deaths outside of a hospital setting; the Ontario Home Care Database for information about provincially funded home care use; and the National Rehabilitation Reporting System for information on in-participant rehabilitation programs.

Participant/caregiver study information will be linked deterministically to the health administrative data using healthcare numbers provided to all individuals insured by the Ontario Health Insurance plan. This will be encrypted for privacy and security. Analysis of linked data will occur within the safe and secure computing environment, and according to the privacy policies of ICES (ICES.on.ca). Study investigators will be provided de-identified summary data.

##### Ambulatory Home Care Record-Hybrid (AHCR-hybrid)

In addition to the AHCR [27], a hybrid form was developed with a set of customized study-specific questions [28] which was appended to better capture the data needs of the planned economic evaluation. We will use the AHCR-hybrid to collect health care resource utilization. Completed monthly, it will be used to capture public and private healthcare utilization including ED visits, hospitalizations, ambulatory/out-patient, and home-based health services. The tool will also capture patient and caregiver private and out-of-pocket health care expenditures, lost productivity relating to caregiving or obtaining medical care as well as adverse events or performance issues related to the use of aTouchAway™ platform and the internet.

Intervention costs for the technology, the aTouchAway^TM^ platform, will be obtained from commercial pricing as well as study data. Both implementation and annual maintenance costs for the intervention will be determined on a per-patient basis and assigned to each participant in the intervention arm.

#### Participant and caregiver experience measures

These will be collected via telephone by a blinded Research Coordinator.

##### Pearlin Self-Mastery Scale

The Pearlin Mastery Scale (PM) measures an individual’s sense and level of mastery. Mastery is a psychological resource defined as “the extent to which one regards one’s life-chances as being under one’s own control in contrast to being fatalistically ruled,” [29]. The 7-item scale comprises five negatively worded items and two positively worded items, presented with the following response options: (1) strongly disagree, (2) disagree, (3) agree, (4) strongly agree. The negatively worded items require reverse coding prior to scoring, resulting in a score range of 7 to 28, with higher scores indicating greater levels of mastery [29]. Caregiver reported Pearlin-Self Mastery Scale was chosen as the co-primary outcome over the self-reported Pearlin-Self-Mastery Scale as not all study participants would be able to complete a self-reported questionnaire. Caregiver reported and participant reported Pearlin Self-Mastery Scales will both be obtained where possible.

##### Zarit Burden Interview (ZBI)

We will use this 22-item questionnaire to assess caregiver burden (change from baseline to 6 and 12 months). The 22 items are assessed on a 5-point Likert scale, ranging from 0 = “never” to 4 = “nearly always.” The questions focus on areas such as caregiver health, psychological well-being, finances, social life, and the relationship between them and their family member (study participant). This questionnaire has demonstrated validity and reliability in caregivers of individuals with chronic conditions [30, 31].

##### Euro-Quality of Life- 5 Dimensions Scale (EQ-5D)

The EQ-5D is a well-known and widely used health status instrument. It provides a concise, generic instrument used to measure, compare, and value health status across disease areas [32, 33]. The instrument provides a health utility score. We will use the EQ-5D-5L (above 18 years of age) and EQ-5D Youth (EQ-5DY) (pediatrics; 4–18 years of age) to assess VAI health status (change from baseline to 6 and 12 months). The EQ-5D will also be used in the cost-utility analysis to observe change between the two groups. The utility score will be used in the calculation of the incremental cost-effectiveness ratio [34].

##### Family Experiences with Coordination of Care (FECC)

We will use the FECC to assess the family perception of the quality of care coordination. This questionnaire has demonstrated validity and reliability in individuals with medical complexity [35]. The FECC will be administered to study participants and family caregivers participating in the qualitative interviews along with other questions that explore experiences with care coordination.

##### VentSS

This questionnaire has been developed for this trial to ask questions about ventilator usage and issues as well as clinical signs and symptoms of the study participants. There are a total of 9 questions.

##### S^3^-NIV

We will use an adapted version of this questionnaire to evaluate 3 important domains related to HMV, specifically respiratory symptoms, sleep quality, and ventilation-related side effects, in both invasively and non-invasively ventilated participants. This is a self-administered questionnaire that has demonstrated validity and test-retest reliability for patients using home non-invasive ventilation ^25^.

#### Care coordination measures

##### Care Coordination Measurement Tool (CCMT)

We will use this validated tool to track health care delivery activities [36]. The tool has established validity for children established on HMV [36]. We will use the tool for HMV center/clinic team members to quantify and characterize all VAI care encounters including time spent [37]. We will assess the relationship between these encounters and later resource utilization. The CCMT will be completed for a randomly selected 10% of study participants in each study arm to minimize the documentation burden for healthcare providers.

#### Process measures

For those participants randomized to the intervention arm, we will measure usage metrics and adherence to TtLIVE intervention components. Specifically, we will document:

1. *n* (%) of the 5 (1, 3, 6, 9, 12 months) clinic visits conducted virtually as opposed to face-to-face
2. *n* (%) of the 5 (1, 3, 6, 9, 12 months) virtual device data downloads available for the HMV team at the virtual clinic visit
3. *n* (%) of participants with a fully completed virtual care plan 6 weeks after study enrolment
4. *n* (%) of the 26 bimonthly symptom/ventilator check-in questionnaires completed (i.e., administered every 2 weeks)
5. *n* (%) of the 12 monthly S^3^-NIV questionnaires completed (i.e., administered monthly)
6. Number of messages, audio calls, and telephone calls initiated by patients/family and healthcare providers
7. Number of concerning (status yellow and red) ventilator monitoring alerts based on bimonthly and monthly symptom monitoring, and time to alert being addressed.
8. Adverse events unique to the use of the aTouchAway™ platform and the internet.

#### Nested qualitative interviews

We will conduct semi-structured interviews with a purposive diverse sample (ALS versus non-ALS, invasive versus non-invasive ventilation, different study sites, rural versus urban residence, low (< 40%) and high (≥70%) intervention adherence) of 20 VAIs, 20 family caregivers of study participants and 20 circle of care healthcare providers (at 6 months (*n*=60)) to explore barriers and facilitators to intervention adherence.

To participate in interviews, participants or their caregivers must be able to communicate verbally for the duration of an interview. Health care providers must meet the following criteria:

##### Interview inclusion criteria for healthcare providers

1. Healthcare provider for individuals newly initiated (in-hospital or outpatient) on HMV
2. Use of the aTouchAway™ for at least five participant encounters
3. Provides informed consent.

Once informed consent is obtained, interviews will be conducted by telephone or via audio or video communication on the aTouchAway™ app based on participant preference. All interviews will be conducted by the same study team member with expertise in qualitative interviewing and knowledge of this study population. Using the same interviewer ensures consistency and opportunity to introduce and probe topics raised by other participants. Interviews are expected to last approximately 60 min, will be digitally recorded with permission, and transcribed verbatim by a professional transcription company. We will remove identifying personal information from interview data prior to the analysis of the interview transcripts.

### Participant timeline {13}

The participant timeline is shown in Table 5.

**Table 5:** Study participant assessment timeline

Tool	Time	Baseline	Every 4 weeks	Monthly	Any clinical encounter	3 month	6 months	12 months
**Participants (with/without caregiver assistance)**								
Demographic/baseline data	10–15 min	X						
Ambulatory and Home Care Record (Hybrid) [27,28 ]	10–20 min	X	X					
Euro-Quality of Life- 5D-5L Dimensions Scale [32,33] (5D-Y for kids)	10–15 min	X					X	X
Qualitative interviews**	60 min						X	X
S3-NIV*** [6]	5 min	X		X				
Pearlin Self-Mastery Scale	5 min	X				X	X	X
VentSS***			X					
**Caregivers**								
Demographic data	5 min	X						
Qualitative Interviews**	60 min						X	X
Family Experiences with Coordination of Care	20–30 min						X	X
Zarit Burden Interview	5–10 min	X					X	X
**Healthcare providers**								
Care Coordination Measurement Tool	< 5 min				X			
Qualitative interviews**	60 min						X	X

***Only completed by intervention group

**Only completed by a subset of participants/ caregivers/ healthcare providers

*Only completed by caregivers completing qualitative interviews

### Sample size {14}

To determine our sample size we used simulations and data from our previous study of VAIs using Ontario health administrative data [2]. We simulated data based on a negative binomial distribution assuming 50% of VAIs visit the ED and had a mean number of 2.6 visits, for an overall yearly incidence rate of 1.3 ED visits. We need 200 participants/ study group to detect a 30% drop-in yearly incidence rate to of 0.91 at 12 months with *α* = 5% and power 80%. Given an estimated attrition rate of 10%, we will recruit a total of 440 participants. For our co-primary outcome, assuming a minimum clinically important difference in the Pearlin Mastery Scale score of 2.95, standard deviation of 4.5, power of 90%, and alpha = 5%, we would require a sample size of 50 study participants in each group for a total of 100 participants [38, 39]. Therefore, with our planned sample size of 440 participants, we are powered for our co-primary outcomes of ED visits and family caregiver reported Pearlin Mastery Scale score.

### Recruitment and patient consent {15}

There are approximately 1200 new ventilators (100/month) across Ontario/year. We are aiming to recruit a conservative 20% (*n*=20) of all new ventilator users/month. Study participants will be recruited from 8 different HMV programs across the province of Ontario. These HMV programs chosen prescribe over 85% of all ventilators in the province. Study recruitment will occur over a 22-month period. The clinical teams from each of the HMV programs will introduce the study.

### Consent or assent {26a}

Potential participants will be introduced to the study by a physician or clinic team member from the participating sites during a clinic appointment, hospital admission, or other patient encounters when HMV prescription occurs. The physician/HMV team member will review the study participant information sheet with the individual and family member. Interested and eligible individuals will be contacted by the unblinded research coordinator (MA) via email or telephone within 0–2 months of HMV initiation to obtain written informed consent.

{26b}

Additional consents for biological specimens are not necessary.

## Assignment of interventions: allocation

### Sequence generation {16a}

Participants will be randomized to the intervention or usual care arms using a centralized randomization schedule through the Ontario Child Health Support Unit (OCHSU). Randomization will be stratified per site. Randomly permuted blocks of size 6 will be used to ensure that the two groups have similar size throughout the trial for each site as well as for the trial overall. We will use a 1:1 allocation ratio stratified by pediatric, adult amyotrophic lateral sclerosis (ALS) patients, adult non-ALS patients, and type of ventilation (invasive or non-invasive).

### Allocation concealment mechanism {16b}

The centralized randomization schedule is embedded into the REDCap study database. When a patient is enrolled, they are assigned to an intervention within the database.

### Implementation {16c}

The allocation sequence is generated using a centralized randomized schedule through the Ontario Child Health Support Unit (OCHSU). The centralized research coordinator (MA) will enroll patients and assign participants to interventions based on the centralized randomization schedule results.

## Assignment of interventions: blinding

### Who will be blinded {17a}

Due to the nature of the intervention, it is not possible for study participants or treating HMV teams to be blinded. The Research coordinator responsible for collecting self-reported outcome measures will be blinded to the allocation assignment. The data analyst will be blinded.

### Procedure for unblinding if necessary {17b}

The design is open label with only outcome assessors being blinded so unblinding will not occur.

## Data collection and management

### Plans for assessment and collection of outcomes {18a}

Study data will be managed (collected and stored) using Research Electronic Data Capture (REDCap), a secure web-based tool for building and managing databases. Patients and family caregivers can complete the study questionnaires over the telephone with a research coordinator (who will input data in the REDCap), or via an electronic link sent via email through REDCap.

The study PI (RA) trained the assessors on the study procedures and tools. In addition, database development and management are being performed in partnership with the Ontario Child Health Support Unit (OCHSU). Data quality routine checks is being performed quarterly by OCHSU and there are standing meetings between the study team and OCHSU to review data quality issues, data check results, and potential data completion issues. In addition, a weekly report is generated by OCHSU for the research team regarding any data completeness/quality issues. Please see Item 12: Outcomes including Table 4 for a description of all the study instruments being used along with their reliability and validity. Data collection forms are available upon request from the study PI (RA).

### Plans to promote participant retention and complete follow-up {18b}

We will provide a CAN$25 voucher for all participants in lieu of their time and an additional $30 voucher for the subset of participants completing qualitative interviews. To promote study retention and increase engagement, in the intervention arm, the unblinded research coordinator will send a weekly message or “tip of the month” through the aTouchAway app. Messages will serve as reminders, and also be clinically relevant to the care of individuals using HMV.

### Data management {19}

All data entry will be done electronically. The research coordinator (MA) will enter data into the database for screening and randomization purposes. Study participants can enter the information directly into REDcap or this will be done by study personnel on their behalf over the telephone. After each data entry timepoint, study personnel reviews the data forms for completion. The OCHSU will develop weekly enrollment and quarterly data quality reports.

### Confidentiality {27}

A REDCap study database will be developed and maintained by the Ontario Child Health and Support Unit (OCHSU), the trial data management center. Participants will be identified in the database by a unique study ID number. Case report forms will also be linked by this ID. An external user interface will be created on REDCap for participants who opt to complete the surveys online. A separate secure list of participant names and contact information will be maintained in an encrypted Microsoft Excel spreadsheet. All study-related electronic data files will be password-protected and reside on the study lead hospital server. Only research team members will have access to the server study file location via password-protected computers.

#### Privacy considerations

aTouchAway™: operates in accordance with Ontario’s Personal Health Information Protection Act (PHIPA) policy. aTouchAway™ is password protected. Data is stored exclusively in a Canadian cloud with no trans-border data transmission. To prevent unauthorized access, maintain data accuracy, and ensure the correct use of information, Aetonix has put in place appropriate physical, electronic, and administrative procedures to safeguard and secure information. We will adhere to all of ICES’ privacy policies and procedures and the privacy best practices endorsed by CIHR (www.cihr irsc.gc.ca/e/290702.html#Summary). All non-ICES study data will be securely stored on OCSHU servers throughout the duration of the study and for up to 10 years after study completion. Anonymized trial data will be available upon completion of the study upon request to enable international prospective meta-analysis.

### Plans for collection, laboratory evaluation, and storage of biological specimens for genetic or molecular analysis in this trial/future use {33}

Please see {26b}; there will be no biological specimens collected.

## Statistical methods

### Statistical methods for primary and secondary outcomes {20a}

The number of ED visits will be analyzed using Poisson or negative binomial regression. Pearlin Mastery Scale scores at 12 months will be analyzed using linear or robust regression models depending on the distribution of the data and adjusting for baseline Pearlin score. Both analyses will adjust for the for the stratified clinical site. We will conduct a secondary analysis of the primary outcome, ED visits, adjusting for a priori chosen clinically important covariates: age, sex, and clinical site; and stratified by pediatric, adult ALS, and adult non-ALS and ventilation type (invasive or non-invasive ventilation). Analysis of secondary outcomes, viewed as exploratory in nature, will vary depending on the outcome: count data (i.e., number of hospitalizations) will be compared with Poisson or negative binomial regression as appropriate; hospital free survival via Kaplan- Meier curves and Cox proportional hazards; time to ED admission and hospitalization using Fine and Gray models to account for competing risk of death; continuous repeated measures outcomes (EQ-5D-3L/5D-Y, FECC, ZBI, and CCMT) using linear mixed effects models.

### Interim analysis {21b}

Interim analyses were not deemed necessary given the low-risk nature of the TtLIVE intervention.

### Methods for additional analyses (e.g., subgroup analyses) {20b}

#### Cost-utility analysis

We will conduct a cost-utility analysis (CUA) to determine the incremental costs (or cost-savings) of the TtLIVE intervention versus usual care for improving quality-adjusted life years (QALYs). A health system and societal approach will be used with a 12-month time horizon. Direct health care costs, including costs of the intervention and health services used during the study period, will be collected from the AHCR-hybrid and by linkage with administrative databases. Costs associated with health care usage as determined in the health administrative data will be used to determine direct health care costs. Direct patient costs will include out-of-pocket expenses attributable to obtaining health care for their HMV. Indirect patient costs will include productivity losses and lost leisure time. Utility will be measured with the EQ-5D-3L and EQ-5D-Y. Cost-effectiveness will be expressed as the incremental cost-effectiveness ratio (ICER), calculated by dividing incremental costs between treatment and usual care arms by the incremental change in QALYs from baseline and 12 months. Costs will be reported in 2023 Can$. Extensive one-way deterministic sensitivity analyses will be performed to evaluate the robustness of the results and to evaluate uncertainty in any assumptions. Probabilistic sensitivity analysis using Monte Carlo simulation will be used to further evaluate uncertainty and establish a point estimate and 95% confidence interval around the ICER.

#### Qualitative interview data analysis

Three researchers (RA, LR, KD) and a research assistant will review transcripts to develop a coding scheme based on recurrent patterns and themes. We will analyze the interviews using directed content analysis [40–44]. We will employ an inductive, four-step content analysis process [45, 46] to identify, code, and categorize important meanings and predominant themes from the text. Following an immersive reading of the transcripts (done iteratively throughout the study), initial patterns and recurring categories will be identified by highlighting sections. The second step will seek similarities and differences between participant accounts. Third and fourth steps involve the creation of codes and their application over the volume of interviews respectively. The larger team will be involved in the in-depth reading of the coding to ensure credibility. Methodological rigor will also be established through prolonged engagement and peer debriefing. Qualitative software (QSR-NVIVO 12) will be employed for thematic grouping and analysis.

### Methods in analysis to handle protocol non-adherence and any statistical methods to handle missing data {20c}

We will use intention to treat analyses as our primary analysis. We will also do a per-protocol analysis. We will describe the amount of missing data. Patterns of missing data will be visually inspected to determine the mechanism of the missing data associations between baseline variables. For the number of ED admissions, we will account for varying lengths of follow-up due to death using the follow-up duration as an offset in the Poisson or negative binomial model. Pearlin score will have a monotonic pattern of missing data and use inverse probability weighting to account for death and dropout. Depending on the amount of missing data for the secondary outcomes, sensitivity analysis will be performed to describe the impact of the missing data.

### Plans to give access to the full protocol, participant level-data and statistical code {31c}

A copy of the full protocol is available from the study PI upon request. The datasets analyzed during the current study will be available within 1 year of completing the trial from the corresponding author and upon reasonable request. The health administrative dataset from this study will be held securely in coded form at ICES and requires special considerations. Legal data sharing agreements between ICES and data providers (e.g., healthcare organizations and government) prohibit ICES from making the dataset publicly available, the full dataset creation plan and underlying analytic code will be available from the study PI upon request understanding that the computer programs may rely upon coding templates or macros that are unique to ICES and are therefore either inaccessible or may require modification.

## Oversight and monitoring

### Data monitoring {21a}

We have established a Trial Steering Committee (TSC) to provide expert oversight for the study to ensure high-quality data and study results. A data monitoring committee was not deemed necessary as the virtual transition intervention was considered a minimal risk to the patients and their families.

### Harms {22}

All adverse events will be reported at the point of occurrence, according to the protocol, to the TSC and subsequently to the SickKids Research Ethics Board (the lead site for the study) if the event is deemed related to the study intervention. Given the nature of the intervention, serious adverse events (SAE) are not anticipated. Potential minor adverse events include delay in communicating with the clinical team because of technical issues related to the app. Of note, these aren’t anticipated to lead to a SAE as the app is not for emergency situation management.

### Auditing {23}

Trial conduct will follow Good Clinical Practice (GCP) guidelines for the safe and effective undertaking of the clinical trial. The Project Management Group will meet weekly to review the trial conduct. Trial Steering Committee meetings will be held every 6 months once recruitment begins. These meetings will review trial progress, discuss barriers to recruitment and retention and potential solutions.

### Protocol amendments {25}

If there are any protocol amendments, the PI will notify each of the 8 HMV centers and a copy of the revised protocol will be sent by the PI to add to the Investigator Site File. In addition, any deviations from the Protocol will be fully documented using a breach report form. All protocol amendments will be updated in the clinical trial registry. Also, all modifications to the protocol will be approved by Clinical Trials Ontario and the Trial Steering Committee.

### Dissemination policy {31a}

Trial results will be disseminated to participants, healthcare professionals, the public, and all relevant stakeholders through community engagement events, presentation of findings at national conferences and symposia, and publication in peer-reviewed journals. We will distribute an executive summary and plain language version of our findings in both French and English. We will partner with Muscular Dystrophy (MD) Canada to highlight this research to the neuromuscular community including a research feature, written pieces on MD Canada’s website and social media. Through the VEP, we have access to their dissemination mechanisms which include a webpage, quarterly newsletter, and annual reporting to the Ministry of Health and Long-Term Care (MOHLTC) in Ontario. Additional input will be sought from knowledge users, both within and outside of the research team to guide our dissemination strategies.

## Discussion

In this study, we will investigate the impact of the TtLIVE intervention delivered through the e-platform aTouchAway™ compared to usual care on healthcare utilization, impact on the patient, family, healthcare costs, and healthcare providers on individuals newly transitioning to HMV. A virtual care platform that offers a comprehensive bundle of virtual care solutions that is sophisticated enough for the complex care demands of the HMV population was not previously in existence. Despite the substantial burden on acute and community healthcare resources, rising population prevalence, profound impact on VAIs and their family caregivers in terms of quality of life and caregiver burden, VAIs and their multiple care transitions remain sub-optimally managed. VAIs are particularly vulnerable due to the new and complicated technology, significant medical complexity, reliance on family caregivers, limited timely access to HMV specialists, and the need to access care across multiple health sectors and disciplines. Therefore, optimization of HMV transitions is imperative. We hypothesize that this virtual transition intervention will reduce emergent healthcare utilization, improve the experience of care, reduce caregiver burden, decrease costs, and enable more efficient use of healthcare provider time.

Strengths of our study design and planned trial include our engagement with stakeholders from the inception of this trial as well as our experience with this intervention through the rollout during the pandemic. Firstly, our trial team consists of patients, family caregivers, community and hospital-based inter-professional clinicians, academics, e-health experts, and knowledge users. Early engagement with stakeholders has iteratively informed the study design to ensure trial output with meaningful results to patients and families as well as the HMV healthcare community. Success of this trial will be facilitated through our collaboration with the Ontario Ventilator Equipment Pool (VEP), our Health System Partner. The VEP provides ventilators to all individuals using HMV in the province. It is funded by the Ministry of Health and Long-term Care (MOHLTC) in Ontario, thereby ensuring rapid dissemination of the trial results to the provincial government. In Canada, healthcare is delivered through the publicly funded provincial systems. Secondly, the pandemic created a novel opportunity to deploy this virtual technology prior to the launch of the trial. In partnership with the Ontario Ventilator Equipment Pool, the Long-term In-Home Ventilator Engagement (LIVE) program was rolled out and provided this e-health intervention to 251 children and adults using HMV to keep them safe and connected at home [47]. As a result, HMV teams across all the study sites have already been trained on the virtual technology and have experience using it for patients. We have also developed and tested, through the LIVE implementation, a supportive onboarding strategy to address challenges with digital literacy. The research team has worked with Aetonix to update challenges/problems with the technology and iteratively improve protocol processes. Participants may experience challenges with the virtual technology’s functionality. The provision of a client success officer that can help participants address these issues by Aetonix, enables us to be more agile in responding to anticipated and unanticipated technical-related issues for the duration of the trial.

We anticipate challenges associated with our trial delivery. First, bi-monthly data collection via telephone may be burdensome for ventilator-assisted individuals and caregivers already dealing with complex health issues. However, our group has demonstrated success in engaging ventilator-assisted individuals and their caregivers using similar data collection methods in the past [6]. Burden will be minimized by flexibility in terms of call scheduling. Second, there is a risk of increased loss to follow-up in the control group because of fewer clinical interactions. To aid retention, the control group will have monthly telephone calls for completion of the AHCR-adapted providing an active opportunity to promote ongoing participant engagement. Third, as with many trials, we anticipate challenges with maintaining recruitment targets. We have actively engaged with our participating sites with bimonthly team meetings throughout the study setup. These meetings will continue throughout the trial to facilitate discussion and input from team members regarding ongoing project conduct, troubleshooting, and strategies to optimize recruitment success. We have included a 3-month recruitment target ramp-up period from trial launch to allow study sites to become proficient with the study procedures. We are also providing tablets with SIM cards to study participants without a smart device and/or reliable internet access to overcome this barrier to participation.

In summary, this trial of a virtual transition intervention for individuals going home with new HMV will provide important data to understand the effects on healthcare utilization, patient and family experience, health system costs, and healthcare provider time due to increased care efficiency. We anticipate our findings will have applicability for the provision of community-based supports for the HMV population in other regions as well as other high-needs populations in the healthcare system.

## Trial status

Patient recruitment began on March 16, 2021. The current protocol version (version 4) is dated (July 9, 2021). Recruitment is estimated to be complete by February 1, 2023.

## Abbreviations

ALS: Amyotrophic lateral sclerosis
CCMT: Care Coordination Measurement Tool
CHEO: Children’s Hospital of Eastern Ontario
CTS: Canadian Thoracic Society
CUA: Cost-utility analysis
ED: Emergency department
EQ-5D: EuroQoL
EQ-5DY: EuroQoL Youth
FECC: Family Experiences with Care Coordination
GP: General Practitioner
HMV: Home mechanical ventilation
HrQoL: Health-related quality of life
ICER: Incremental cost-effectiveness ratios
IC/ES: Institute of Clinical Evaluative Science
NIV: Non-invasive
NP: Nurse Practitioner
OCCI: Ontario Case Costing Initiative (Cost Analysis Tool)
OCHSU: Ontario Child Health Support Unit
OHIP: Ontario Health Insurance Plan
QALYs: Quality-adjusted life years
RCT: Randomized controlled trial
REB: Research Ethics Board
REDCap: Research Electronic Data Capture
RT: Respiratory Therapist
SPSS: Statistical Product and Service Solutions
TSC: Trial Steering Committee
VAI: Ventilator-assisted individuals
VEP: Ventilation equipment pool
VPN: Virtual Private Network
ZB: Zarit Burden
